# Mobile Augmented Screening to Increase HIV Testing Among Emergency Department Patients as Young as 13 Years

**DOI:** 10.7759/cureus.15829

**Published:** 2021-06-22

**Authors:** Ian D Aronson, Jingru Zhang, Sonali Rajan, Mona Bugaighis, Lisa A Marsch, Mobolaji Ibitoye, Lauren S Chernick, Don C Des Jarlais

**Affiliations:** 1 Research, Digital Health Empowerment, Brooklyn, USA; 2 Social and Behavioral Sciences, New York University School of Global Public Health, New York, USA; 3 Measurement and Evaluation, Teachers College, Columbia University, New York, USA; 4 Department of Health and Behavior Studies, Teachers College, Columbia University, New York, USA; 5 Emergency Medicine, Columbia University Irving Medical Center, New York, USA; 6 Center for Technology and Behavioral Health, Dartmouth College, Lebanon, USA; 7 Institute for Population Research, The Ohio State University, Columbus, USA; 8 Epidemiology, New York University School of Global Public Health, New York, USA

**Keywords:** hiv, technology, adolescent, emergency department, youth

## Abstract

Because adolescents and emerging adults are frequently not offered HIV testing, and often decline tests when offered, we developed and tested a tablet-based intervention to increase HIV test rates among emergency department (ED) patients aged 13-24 years. Pediatric and adult ED patients in a high volume New York City hospital (N = 295) were randomized to receive a face-to-face HIV test offer, or to complete a tablet-based intervention that contained an HIV test offer delivered via computer. Test rates in both conditions were then compared to historic test rates in the same ED during the previous six months. Among participants aged 19 years and younger who were offered HIV testing and declined before enrollment in the study, participants in the tablet-based condition were 1.7 times more likely to test for HIV compared to participants in the face-to-face condition. Participants aged 19 years and younger were three times as likely to test for HIV compared to patients the same age who were treated in the previous six months (26.39%, n = 71 study participants vs. 10.29%, n = 189 prior patients, OR = 3.13, $\chi$^2 ^= 54.76, p < 0.001). Protocols designed to offer HIV testing to all eligible patients can significantly increase adolescent test rates compared to standard practice. Because tablets are equally effective compared to face-to-face offers, and in some cases more so, EDs may consider tablet-based interventions that require fewer staff resources and may integrate more easily into high-volume workflows.

## Introduction

The Centers for Disease Control and Prevention (CDC) and the United States Prevention Services Task Force, and the American Academy of Pediatrics all recommend routine HIV testing for adolescent patients in healthcare settings, including emergency departments (EDs), with at least annual re-testing for patients who engage in behaviors that increase HIV risk or live in areas with high HIV prevalence [1,2]. Additionally, New York State requires offering HIV testing “as a part of routine health care to all individuals age 13 and older” [3]. However, most ED patients are not offered HIV testing [4,5] and many adolescents and emerging adults, including those at increased risk, have not been tested in any setting, much less re-tested [6].

The majority of sexually active high school students have never tested for HIV [7,8], large proportions of adolescent sexual minority males who engaged in condomless sex at last intercourse report never testing for HIV [9], and half of young HIV positive men who have sex with men remain undiagnosed [10]. When youth do test for HIV, current ED standards of care do not include education about prevention and retesting for adolescents who test negative yet engage in high-risk behaviors [11]. Dangerous gaps in HIV-related health literacy have also been documented among adolescents, including a lack of understanding that people who test negative may need to re-test if they have been recently exposed to HIV [12] and that many HIV positive youth do not know their status [13]. Similar literacy deficits may potentially lead youth to incorrectly believe they do not require testing because they are not at risk [14], and therefore to decline testing when offered.

EDs offer important points of contact for young people who do not receive adequate opportunities to test for HIV or learn about the importance of prevention and testing because they lack access to primary care. EDs also present opportunities to test youth who do have access to testing but have previously elected not to test because they do not recognize their potential HIV risk. Unfortunately, adolescent ED patients who decline HIV testing are rarely, if ever, offered a second chance to test during the same ED visit. Further, young patients are often hesitant to report risk behaviors or even discuss HIV-related issues with medical staff [15,16], and staff are often uncomfortable discussing sexual behavior with young patients [16], especially with sexual minority adolescents [17]. Young ED patients, especially those accompanied by their parents, are unlikely to initiate requests for HIV testing [18]. Thus, if ED staff do not offer testing to all eligible patients, including those who do not report risk, they may regularly miss key opportunities to address undiagnosed HIV among youth.

Digital technology offers potential solutions. Our team has created brief (<15 minutes) technology-based interventions to increase HIV testing in high-volume ED settings [19-21]. Our interventions have been shown to increase HIV testing [19], including testing among patients who initially decline tests offered by hospital staff [20]. Our interventions have also been shown feasible and acceptable to participants, including ED patients aged 18-24 [21], and to ED staff [22]. However, our previous trials did not include participants younger than 18 years, nor did they include direct comparisons between our technology and face-to-face offers of HIV testing. Also, because previous interventions did not enable contact with participants post-ED discharge, we could not collect follow-up data. Additionally, a recent systematic review shows very few technology-based interventions were developed to increase youth HIV testing in clinical settings and evaluated via randomized controlled trials; none of these included participants younger than 15 years [23].

To increase testing among participants as young as 13 years, in keeping with CDC guidelines [2] and New York State Law [3], and to improve upon the design limitations described above, we created a new tablet-based intervention, the mobile augmented screening (MAS) tool, and then conducted an effectiveness trial in one of the highest volume emergency departments in New York City. Patients were eligible to participate if they had not already accepted the offer of an HIV test. This included patients who had not yet been offered an HIV test by ED staff, as well as participants who were offered an HIV test and declined. Participants were randomized to receive a face-to-face test offer from ED personnel, or to receive an HIV test offer via the MAS. The face-to-face test offers were standardized for all participants, and are described in greater detail in the Methods section. Key components of the MAS include an iteratively developed video designed to address foundational gaps in HIV-related health literacy identified by our research and others (e.g., Swenson et al. [12]), paired with measures designed to document baseline HIV-related health literacy levels and potential improvement after watching the video. To enable post-discharge communication with participants, the MAS contains a text message component that emphasizes the importance of ongoing HIV prevention, and encourages re-testing as needed after 90 days. The texts also facilitated remote evaluations of participants’ HIV literacy levels 4, 8, and 12 weeks after their ED visit.

The current research was designed to answer the following research questions:

Feasibility

Would we be able to recruit patients aged 13-24 years in a high volume, urban ED?

Would participants complete the intervention, and if so, would participants agree to receive follow-up text messages and provide mobile phone numbers?

Acceptability

Would participants give the MAS positive or negative ratings using a set of automated acceptability scales used by our team in prior NIH-funded research?

Effectiveness

Would the MAS improve HIV-related health literacy among participants, and if so, would literacy improvements persist over time?

Would the MAS increase HIV test rates among participants aged 13-24 years compared to a face-to-face offer of HIV testing, and to historical test rates within the ED where the trial was implemented?

## Materials and methods

From July 2019 through early March 2020, research assistants (RAs) recruited a convenience sample of patients from the pediatric and adult EDs at a large, tertiary-care academic hospital in New York City that annually serves 50,000 and 100,000 patients, respectively. Patients aged 19 and younger are treated in the pediatric ED, patients aged 20 and older are treated in a separate adult ED.

Procedure

RAs approached patients after they had already been seen by a triage nurse and examined by an ED physician. Patients were eligible to participate if they were aged 13-24 years, not known to be HIV positive, not critically ill (at the discretion of the attending physician), and not a prisoner. Before approaching potential participants, the RA reviewed each patient’s electronic health record (EHR) to determine whether staff had offered HIV testing, and if so, whether the offer was accepted or declined. If the RA could not confirm a prior HIV test offer from EHR data, the RA asked the patients’ physician.

RAs were instructed to approach all eligible patients. Participants were recruited in the areas where they were receiving treatment and did not receive incentives to participate. RAs obtained written informed consent from all patients age 18 years and older and written parental consent and patient assent for all patients younger than 18. All procedures, consent forms, and study materials were approved by all governing IRBs.

All participants used the MAS to enter basic demographic data, including age, race, and gender. MAS software then randomly assigned participants into one of two conditions, using a proprietary algorithm designed by the study PI and a colleague to ensure even distribution of participants.

MAS condition

MAS condition participants remained in the area where they were receiving treatment and used the MAS to report their HIV testing history, answer pre-test HIV-related health literacy questions, watch a five-minute video designed to address barriers to youth HIV testing [24], and answer post-test health literacy questions. After MAS condition patients responded to post-test questions, the MAS asked each patient if they would like an HIV test. Possible responses were yes or no. After patients responded, the MAS prompted participants to enter a mobile phone number. The MAS explained that participants who provided a phone number would receive text messages once a week for 12 weeks, that some of the messages would contain links to surveys, and participants who responded to survey questions would earn $25 gift cards. The MAS allowed participants to advance to the next screen whether or not they entered a mobile phone number. The MAS then presented a set of acceptance items used in previous NIH-funded research (e.g. [25,26]).

Face-to-face condition

The face-to-face condition was designed to meet an exceptionally high standard of care for HIV test offers. The RA escorted the participant out of the treatment area away from family members and other patients. The RA then handed the participant a printed flyer produced by the New York State Department of Health titled “Say yes to the HIV test” [27]. Bullet-point text on the flyer explains that HIV can be spread via unprotected sex, sharing needles, childbirth, or breastfeeding, and notes that treatment is available for those who test positive. Large text at the bottom of the flyer states “We’re asking everyone. It’s the law.” The RA then offered these participants an HIV test.

Historical data

To compare post-intervention HIV test rates in both conditions to historic HIV test rates in the pediatric and adult EDs where the trial was implemented, our team examined health record data from patients who visited the EDs in the six months prior to data collection (January 2019-July 2019). These records indicated whether patients had been offered HIV testing and declined, offered HIV testing and accepted, or had not been offered HIV testing. No HIV test results were included.

The historical comparison data included records from patients with eligibility criteria comparable to those in our trial. The historical comparison was limited to patients who visited the ED Monday through Friday, 9 am to 5 pm, the timeframe in which most patients were recruited for the current study. Prior to analysis, the comparison dataset was cleaned of identifying information. Records were removed for patients who were transferred to another hospital or who left before being treated, in case patients had not been offered HIV testing due to shorter ED visits. Records that contained conflicting information about whether patients had accepted or declined HIV testing were also removed.

Data analysis

Analyses were conducted with R (version 4.0.0). A series of chi-square tests were used to compare participant responses by treatment group. Non-parametric tests were used to compare test rates among the trial sample to test rates among the historic test sample of ED patients.

## Results

Feasibility

Our team enrolled 295 participants, approximately 77% (n = 226) self-identified as Hispanic or Latino including 32.5% (n = 96) Black Latino, 14.5% (n = 43) White Latino, 11% Native Hawaiian or other Pacific Islander (n = 31), 9% more than one race (n = 26), 8% American Indian (n = 25), and 2% Asian (n = 5). Approximately 10% (n = 30) identified as Black or African American non-Latino, and 5% (n = 15) White non-Latino. Approximately 8% (n = 24) identified as non-Latino multiracial. See Table 1 for details on age and gender.

**Table 1:** Age and gender of participants (N = 295).

Age	Male	Female	Trans male	Trans female	Genderqueer	Additional category	Declined to state
13	11	15	1		1		
14	13	14				1	
15	22	17					
16	6	22		1			
17	12	16		1			
18	19	38					
19	20	38				1	
20	1	4					
21	1	2					
22	4	1					
23	1	5					
24	0	6			1		
Total	110	178	1	2	2	2	

Among our sample, 147 participants were randomized into the MAS condition and 148 into the face-to-face condition. Roughly half of the participants (n = 153) were younger than 18 years, approximately one-third (n = 95) were younger than 15 years. We ended recruitment in early March 2020 due to the New York City coronavirus outbreak.

All 147 MAS participants completed the full intervention and answered all questions. Approximately 85% (n = 122) provided a mobile phone number and agreed to receive follow-up text messages. Of these, approximately 61% (n = 74) responded to MAS text messages by submitting responses to at least one online follow-up survey.

Acceptability

Participants reported the MAS was exceptionally easy to understand (9.26 out of a possible 10) and use (9.29 out of 10). Participants also reported the MAS was highly useful (8.76 out of 10). For more detail, see Table 2.

**Table 2:** Acceptability scores (measured on a scale of 1 to 10)

Question	Mean score	SD
How interesting was the program you just completed?	8.14	2.05
How useful was the program you just completed?	8.75	1.76
How much new information did you learn as a result of the program you just completed?	8.14	2.16
How easy to use was the section of the program you just completed?	9.29	1.47
How much did you understand the program you just completed?	9.26	1.35
How much did you like the program you just completed?	8.76	1.67
How threatening did you find the program you just completed?	3.35	3.38

Effectiveness

HIV-Related Health Literacy

Pre- post-tests of HIV-related health literacy among intervention group participants show increases on all four items. As detailed in Table 3, pre- post- increases on three of the four items were statistically significant. Online health literacy tests conducted 4, 8, and 12 weeks after the initial intervention show literacy levels remained near or above post-test levels during the full 12-week follow-up period.

**Table 3:** HIV-related health literacy test questions and results, pre-post. *Indicates significance at the p < 0.05 level. **Indicates significance at the p < 0.001 level.

Question	Pre-test percent correct	Post-test percent correct	Week 4 percent correct	Week 8 percent correct	Week 12
Under some circumstances a person who tests negative for HIV may need to re-test in 90 days.	74.83	95.24	92.24	89.09	92.68
		t (146) = -5.47			
		p = 1.908e-07**			
Drinking alcohol before sex or smoking weed before sex can increase a person’s risk of HIV infection.	26.53	68.03	57.58	67.27	70.73
		t (146) = -9.20			
		p = 3.48e-16**			
60% of young people who have HIV don’t know.	88.44	95.92	98.49	98.19	95.12
		t (146) = -2.44			
		p = 0.02*			
People can be infected with HIV for years before they show symptoms.	82.99	85.71	95.46	87.27	95.12
		t (146) = -0.75			
		p = 0.45			

HIV tests offered

Analyses of test offers show two-thirds of participants (n=200) had not been offered an HIV test during the ED visit in which they enrolled in our study. Considerably fewer patients in the pediatric ED had been offered HIV testing prior to joining our study compared to participants in the adult ED. See Table 4 and Table 5 for details.

**Table 4:** Participants not offered HIV testing during study ED visit, by age (both treatment conditions). ED: emergency department.

Age	Not offered testing
13	21 (75%)
14	18 (64%)
15	30 (77%)
16	19 (66%)
17	22 (76%)
18	35 (51%)
19	41 (69%)
20	2 (40%)
21	2 (67%)
22	2 (40%)
23	3 (50%)
24	5 (71%)
Total	200 (68%)

**Table 5:** Lifetime test history (only MAS condition participants, n = 147, were asked) MAS: mobile augmented screening.

Age	Never offered HIV testing
13	10
14	13
15	12
16	7
17	5
18	7
19	9
20	1
21	0
22	0
23	0
24	1
Total	65

HIV test acceptance

Overall, approximately 26% of participants tested for HIV post-intervention. Among all patients enrolled in the trial, there was no difference in the percentage of participants who accepted an HIV test via the MAS (n = 39 out of 147, 13.22%) compared to those who accepted in the face-to-face condition (n = 39 out of 148, 13.22%, $\chi$^2^ = 0, p = 1).

Differences emerged between participants who declined HIV tests offered by ED staff immediately prior to enrolling in our study, and participants who had not been offered HIV testing by hospital staff during the ED visit in which they enrolled in our study. Participants who were offered HIV testing by staff during their current ED visit and declined prior to joining our study (n=95) were slightly more likely to test if they were randomly assigned to the MAS condition (n = 12 out of 52, 23.08 %, compared to n = 9 out of 43, 20.93% of participants in the face-to-face condition, OR = 1.13, $\chi$^2 ^= 6.835e-06).

The observed effect becomes notable among pediatric patients. Among trial participants aged 19 and younger who initially declined testing offered by hospital staff (n = 83), participants assigned to the MAS condition were 1.7 times more likely to accept an HIV test offer compared to those assigned to the face-to-face condition (24.44%, 11 out of 45 of those in the MAS condition accepted an HIV test offered via tablet computer, while 15.79%, or 6 out of 38 of those in the face-to-face condition accepted an HIV test offered by an RA).

In contrast, patients aged 19 and younger who had not been offered HIV testing by hospital staff prior to enrollment in our study (n=186) were equally likely to test regardless of the treatment group into which they were enrolled (29.07%, or 25 out of 86, participants in the MAS condition accepted HIV testing offered via tablet computer versus 29.00%, or 29 out of 100, participants in the face-to-face condition who accepted an HIV test offered by an RA).

Of those who accepted, all were tested by ED staff, with the exception of 6 participants whose medical records indicate HIV test orders were cancelled by ED staff prior to testing. There were no positive HIV tests among trial participants.

Historic data

Records show that in the 6 months prior to the start of our trial, out of a total 3,422 ED patients aged 13-24 years and eligible for HIV testing: 13.41% (n = 459) tested for HIV; 55.64% (n = 1904) were offered HIV testing and declined, and 30.95 % (n = 1059) were never offered HIV testing.

Among those treated in the pediatric ED (patients aged 19 years and younger, n = 1837): 10.29% (n = 189) tested for HIV; 52.80 % (n = 970) were offered HIV testing and declined, 36.91 % (n = 678) were never offered HIV testing.

Historic test rates compared to current trial

Overall, participants in the MAS trial (n = 295) were more than twice as likely to test for HIV post-intervention compared to patients of the same age range treated in the same ED (n = 3,422) in the six months prior to our study (26.44 %, n = 78 of trial participants versus 13.41%, n = 459 of prior participants, OR = 2.32, $\chi$^2^ = 36.25, p = 1.74e-09).

When test rates are analyzed only among those aged 19 years and younger, participants in our trial were more than three times as likely to test for HIV compared to those treated in the same ED in the six months prior (26.39%, n = 71, of trial participants compared to 10.29%, n = 189, of prior patients, OR = 3.13, $\chi$^2^ = 54.76, p = 1.359e-13).

## Discussion

The central goals of the current study were to increase testing among adolescent and young adult ED patients, and to examine how different types of test offers (MAS versus face-to-face) would influence participants’ decisions to accept testing. Findings indicate our efforts were both feasible and highly acceptable. Not only did all MAS participants complete the full intervention, but participants gave the MAS very high acceptability scores in all categories.

More importantly, a review of patient test offers and test acceptance rates (including patients enrolled in our trial and patients in the ED prior to the start of our data collection) underscores the need for this type of intervention. Adolescent patients are frequently not offered HIV testing. Introducing a protocol designed to ensure HIV testing is offered to all eligible patients, whether face-to-face or via tablet-based intervention, can significantly increase HIV test rates. Overall, study participants tested for HIV at more than double the rate of patients in the same ED during the prior six months. Among pediatric patients, study participants were 3.1 times more likely to test. Although few, if any, EDs would have the ability to devote a full-time staff member to privately offer HIV tests to all eligible ED patients as we did in the face-to-face condition, the MAS is a self-contained intervention designed so it can be administered by a student or volunteer without interrupting workflows or hiring additional staff.

Additionally, our findings show that the method by which testing is offered can influence outcomes. Among patients aged 19 years and younger, who the current study shows are substantially less likely to be offered HIV testing compared with patients aged 20-24 years, those who initially decline HIV tests offered by hospital staff are 1.7 times more likely to accept an HIV test offered by computer compared to those offered face-to-face. Our studies have previously shown that tablet-based interventions can increase HIV testing by ED patients who initially decline tests offered by hospital staff. However, it remained to be seen whether the increase was due to the tablet-based intervention or simply to the second offer of an HIV test. Based on findings from the current study, EDs may consider making face-to-face HIV test offers to all eligible patients at triage, and then administering the MAS or a similar intervention to patients who decline the first offer.

Results showing highly significant increases on three out of four health literacy test items appear similarly promising. These items were designed not only to measure participant understanding of key points, but in combination with the iteratively developed MAS intervention video [28], were intended to ensure participants had a solid enough grasp of HIV testing-related issues to make informed decisions when presented with a test offer. Some care providers interviewed by our team [29] noted testing could be improved by delivering basic education, and one physician suggested it might be inappropriate to offer testing without relevant education. Findings that our materials appear to have significantly increased participants’ HIV literacy, and that follow-up health literacy scores remain above pre-test levels 12 weeks later, may help address these provider concerns.

Limitations

A primary limitation of our study is the parental consent requirement. Young patients frequently come to an ED with sexual and reproductive health complaints unaccompanied by a parent or guardian. They are an important population group to reach with interventions to increase HIV testing and related health literacy, yet due to the parental consent requirement (required by the hospital IRB) we were unable to include them in the present study. Additional research is warranted with unaccompanied minors.

An additional important limitation is the lack of a non-intervention control condition. Comparisons to data from the same ED in the six months prior to the current study indicate both trial conditions significantly raised participant test rates. Due to the importance of youth HIV testing, our study team did not want to offer a control condition that would not encourage participants to test. We, therefore, instructed RAs to bring participants in the face-to-face condition to a private area before offering HIV testing, and in doing so we created an additional intervention condition. Based on results from the current study, we hypothesize the MAS would greatly increase test rates compared directly to standard ED operating procedures, and additional research is now warranted comparing the MAS to standard care.

## Conclusions

Our findings indicate that among participants who initially decline HIV testing, the MAS is more effective compared to face-to-face offers. Overall, the MAS is just as effective as having a person privately offer HIV testing to all eligible patients. Given these data, it appears EDs can use tablet-based interventions to increase HIV test rates among adolescent and emerging adult ED patients who might otherwise not be offered opportunities to test, and especially among those who decline tests offered face-to-face by hospital staff.
